# Mimicking Natural Photosynthesis: Designing Ultrafast Photosensitized Electron Transfer into Multiheme Cytochrome Protein Nanowires

**DOI:** 10.3390/nano10112143

**Published:** 2020-10-28

**Authors:** Daniel R. Marzolf, Aidan M. McKenzie, Matthew C. O’Malley, Nina S. Ponomarenko, Coleman M. Swaim, Tyler J. Brittain, Natalie L. Simmons, Phani Raj Pokkuluri, Karen L. Mulfort, David M. Tiede, Oleksandr Kokhan

**Affiliations:** 1 Department of Chemistry and Biochemistry, James Madison University, Harrisonburg, VA 22807, USA; marzolf.4@buckeyemail.osu.edu (D.R.M.); mckenzie4@wisc.edu (A.M.M.); coleman.swaim@quinnipiac.edu (C.M.S.); brittatj@dukes.jmu.edu (T.J.B.); 2 Chemical Sciences and Engineering Division, Argonne National Laboratory, Lemont, IL 60439, USA; ponomarenko@anl.gov (N.S.P.); mulfort@anl.gov (K.L.M.); tiede@anl.gov (D.M.T.); 3 Department of Biology, James Madison University, Harrisonburg, VA 22807, USA; simmo2nl@dukes.jmu.edu; 4 Biosciences Division, Argonne National laboratory, Lemont, IL 60439, USA; prp0015@auburn.edu

**Keywords:** biohybrids, biomimetic photosynthesis, photo-induced electron transfer (PET), Ultrafast electron transfer (ET), PpcA, cytochrome c_7_, Ruthenium tris(bipyridine)

## Abstract

Efficient nanomaterials for artificial photosynthesis require fast and robust unidirectional electron transfer (ET) from photosensitizers through charge-separation and accumulation units to redox-active catalytic sites. We explored the ultrafast time-scale limits of photo-induced charge transfer between a Ru(II)tris(bipyridine) derivative photosensitizer and PpcA, a 3-heme c-type cytochrome serving as a nanoscale biological wire. Four covalent attachment sites (K28C, K29C, K52C, and G53C) were engineered in PpcA enabling site-specific covalent labeling with expected donor-acceptor (DA) distances of 4–8 Å. X-ray scattering results demonstrated that mutations and chemical labeling did not disrupt the structure of the proteins. Time-resolved spectroscopy revealed three orders of magnitude difference in charge transfer rates for the systems with otherwise similar DA distances and the same number of covalent bonds separating donors and acceptors. All-atom molecular dynamics simulations provided additional insight into the structure-function requirements for ultrafast charge transfer and the requirement of van der Waals contact between aromatic atoms of photosensitizers and hemes in order to observe sub-nanosecond ET. This work demonstrates opportunities to utilize multi-heme c-cytochromes as frameworks for designing ultrafast light-driven ET into charge-accumulating biohybrid model systems, and ultimately for mimicking the photosynthetic paradigm of efficiently coupling ultrafast, light-driven electron transfer chemistry to multi-step catalysis within small, experimentally versatile photosynthetic biohybrid assemblies.

## 1. Introduction

The magnitude of the challenge needed to achieve efficient atmospheric CO_2_ utilization has led to renewed interest in artificial photosynthetic approaches for CO_2_ capture and conversion [1,2,3,4,5]. Photosynthesis provides a paradigm for the efficient coupling of ultrafast, light-driven electron transfer (ET) chemistry to slower, multi-step, water-splitting catalysis [6,7,8,9,10]. Key questions remain whether photosynthesis could be re-designed to achieve comparably efficient coupling of light-exited states to CO_2_ reduction and conversion to liquid fuels [11]. Multiple approaches have been developed for artificial photosynthesis. Currently one of the most effective systems is based on the introduction of artificial catalysts within native photosynthetic reaction center protein complexes [12,13]. However, the size and complexity of these systems make their rational optimization and development challenging. On the other hand, minimalistic approaches have their own share of issues. In particular, there are overwhelming challenges associated with stabilization of charge separated states long enough to accumulate a sufficient number of electrons for catalysis resulting in a limited number of turnovers [14,15,16,17,18]. 

While there is an extensive literature on the photophysics and photochemistry of photo-induced charge separation involving single redox cofactor proteins such as cytochrome (cyt) c, azurins, myoglobin, etc. [19,20,21,22,23,24,25,26,27,28,29,30,31,32], because of their ability to store only one electron these designs are not necessarily optimal for multi-electron catalysis. Furthermore, ET rates for the covalently linked photosensitizer-redox protein biohybrids reported to date have been on the 10 ns timescale or slower [20,21,23,24,25,28,29,30,31,33,34,35]. A number of hypotheses to test the mechanisms of ET in these biohybrids have been developed including packing density [36,37], pathway tunneling [38,39,40,41], superexchange [42,43], and a number of quantum mechanical approaches [44,45,46,47,48,49,50]. However, ultrafast light-induced electron transfer that is ultimately coupled to charge-accumulation has not been demonstrated with biohybrid designs based on single redox cofactor proteins. Use of longer excited state lifetimes, and in particular electron injection from the triplet states, significantly increases the chance of production of reactive oxygen species, which leads to rapid degradation of artificial photosynthetic complexes. A key challenge lies in developing biohybrids that mimic photosynthetic designs by utilizing ultrafast primary electron transfer to achieve efficient charge separation, followed by sequential secondary electron transfer reactions for long-lived charge separation and charge accumulation. 

To address these challenges and leverage biological diversity and evolutionarily optimized structures, we have investigated a biohybrid approach that combines artificial photosensitizers with biological “nanowires”—proteins carrying multiple redox cofactors that potentially can serve as an electron transfer chain for sequential electron transfer and charge accumulation. Here we report on an investigation of photo-induced charge transfer between a Ru(II)(bpy)_3_ (bpy = 2,2′-bipyridine) derivative covalently linked to cysteine residues on PpcA, a triheme 71 amino acid c-type cytochrome with a diameter of about 2.5 nm. The ruthenium photosensitizer-PpcA constructs are used to investigate opportunities to introduce ultrafast, photo-induced charge injection into this model multi-heme system. In our previous report we demonstrated that the K29C mutant of PpcA showed charge separation and recombination time constants of 6.4 ps and 38 ps, respectively—approximately 1000-fold faster than any other artificial covalent protein-photosensitizer system [51]. However, the structural requirements for such a fast kinetics were not completely clear. One possible explanation was that K29C mutation introduces a covalent attachment site within the Heme-I binding motif (C_27_K_28_K_29_C_30_H_31_), where C27 and C30 are the heme thioether attachment points and H_31_ is the axially coordinating histidine, providing a pathway consisting of 11 covalent bonds separating aromatic atoms on the Ru(II)(bpy)_3_ derivative photosensitizer and heme electron acceptor. However, depending upon the conformation of the linked photosensitizer-PpcA construct, through-space electron transfer pathways may also exist. We note that the K29C site shows a strong selectivity for covalent linkage, selecting the Δ-enantiomer out of a racemic mixture of a Ru(II)(bpy)_3_ derivative [52], implying a precise 3D geometry for the linked complex and close spatial positioning to an adjacent Heme-III [51]. A comparison of photosensitizer-to-heme ET using linkage sites within c-type heme binding motifs provides opportunities to compare the dynamics for sites that are similarly bracketed by heme cofactor thioether linkages and thus have common through-bond pathways, but can be anticipated to differ in through-space configurations. 

**Figure 1:**
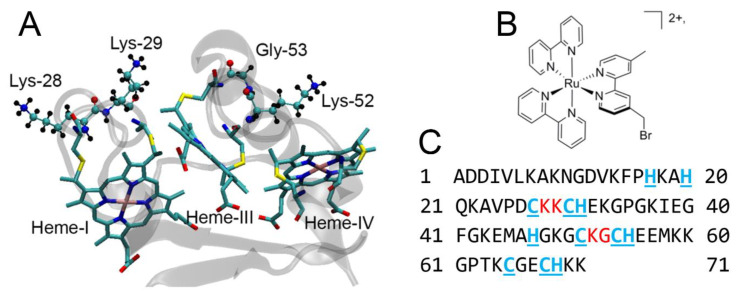
(**A**) Structure of PpcA, a 3 heme c-type cytochrome from *Geobacter sulfureducens* showing orientations of Lys-28, Lys-29, Lys-52, and Gly-53 residues with respect to the heme groups. (**B**) structure of Ru(II)(bpy)_2_(4-bromomethyl-4′-methyl-2,2′-bipyridine, the photosensitizer used in this work. (**C**) sequence of PpcA showing in underlined blue positions of heme ligands and in red locations of cysteine mutations.

Here we test a hypothesis that a covalent photosensitizer attachment to a residue within the heme binding motif sequence (CXXCH) will result in sub-nanosecond photo-induced charge transfer rates. In addition to the previously characterized K29C mutant, we developed, expressed, and characterized three more PpcA mutants, K28C, K52C, G53C, with cysteine attachment sites introduced within the CXXCH heme binding motifs for Heme-I, described above, and for the comparable sites within the binding motif for Heme-III, C_51_K_52_G_53_C_54_H_55_, illustrated in Figure 1. While K52C indeed showed ultrafast charge transfer kinetics (2.3 ps and 5.9 ps for apparent charge separation and recombination, respectively), the K28C and G53C mutants showed dynamics about 3 orders of magnitude slower. Our results show that PpcA can serve as an acceptor, harvesting electrons from excited photosensitizers on the ultrafast timescale, and the factor controlling the rate of charge transfer is the tertiary structure of the biohybrids and the strength of structural coupling between photosensitizers and protein frameworks. 

## 2. Materials and Methods

Ru(II)(bpy)_2_(4-bromomethyl-4′-methyl-2,2′-bipyridine)·2PF_6_, Ru(II)(bpy)_2_(Br-bpy), was synthesized following a previously published procedure [53]. Proton nuclear magnetic resonance (^1^H NMR) was performed on a Bruker DMX500 (Billerica, MA, USA) and referenced to tetramethylsilane (Sigma-Aldrich, St. Louis, MO, USA) or residual solvent peak. Electrospray ionization mass spectrometry (ESI-MS) data were collected on a ThermoFisher LCQ Fleet (Waltham, MA, USA), from dilute acetonitrile solution in positive ionization mode. All characterization results matched previously reported values. 5-α and BL21 (DE3) *E. coli* cells were purchased from New England Biolabs (Ipswich, MA, USA). All chemicals were purchased from Fisher Scientific (Waltham, MA, USA), unless specified otherwise.

New cysteine mutations in PpcA were introduced to a template pVA203 plasmid [54], as described previously [51] using primer oligonucleotides (Invitrogen) summarized in Table S1. Mutant DNA sequences were verified at the University of Chicago DNA Sequencing and Genotyping Facility. Mutant forms of PpcA were expressed in BL21 (DE3) *E. coli* strain containing *c*-type cytochrome maturation genes (Ccm) in a pEC86 vector [55] and purified as reported previously [56]. In short, cell pellets were resuspended in 1:20 (*w/v*) buffer (20 mM Tris, pH 7.5, 20% (*w/v*) sucrose, 0.5 mM EDTA), 40 mg/L lysozyme) and shaken for 30 min at room temperature. Cell suspensions were centrifuged at 6500× *g* for 30 min. The resulting supernatant fractions (“periplasm”) were collected and dithiothreitol (DTT) was added to a final concentration of 2 mM. After incubation of at least 3 min, periplasmic fractions were loaded to a chromatography column packed with Macro S ion exchange resin (Bio-Rad) pre-equilibrated in 20 mM Tris, pH 7.5 (“Buffer A”). After loading of periplasm, the column was washed with 5 column volumes of Buffer A supplemented with 1 mM DTT. PpcA was eluted with Buffer A with 200 mM NaCl and 1 mM DTT. The first eluted intensely red-colored band was collected and concentrated using Amicon centrifugal filters with a cutoff of 3 kDa to the final concentration of 1–2 mM. Stock PpcA concentrations were determined as previously described [57]. 

The molecular mass for all of the expressed cysteine mutants, as well as the products following Ru(II)(bpy)_2_(Br-bpy) labeling described below, were analyzed using liquid chromatography (LC)/ESI-MS. The liquid chromatography used a 2–40% MeCN linear gradient in water with 0.1% formic acid on a C18 Discovery Bio analytical high-performance liquid chromatography (HPLC) column (Sigma-Aldrich) installed on an Agilent 1100 HPLC system (Santa Clara, CA, USA). The eluant was analyzed with a photodiode array detector spectrophotometer and ESI-MS (Agilent 6530 qTOF). 

Labeling of reduced cysteine mutants of PpcA (0.5–1.0 mM) with Ru(II)(bpy)_2_(Br-bpy) followed the previously described procedure [51]. Here, the labeling of mutant PpcA was performed at room temperature in 10 mM Tris, pH 7.5 buffer with 100 mM NaCl with a 5-fold molar excess of Ru(II)(bpy)_2_(Br-bpy) added from a 50 mM stock solution in *N*,*N*-dimethylformamide After overnight gentle shaking in a vial shielded from light, samples were centrifuged to pellet particulates and the supernatant was applied to a Sephadex G25 column to separate unreacted labeling reagent from the protein. A red cytochrome band showed a clear separation from an orange band of unreacted Ru(II)(bpy)_2_(Br-bpy). The covalent attachment of the label to the protein was verified with HPLC-MS as described above.

Redox titrations were performed in a glovebox deoxygenated by purging nitrogen for at least 30 min until the oxygen content was under 0.1%. Cytochrome samples were first exchanged to 100 mM potassium phosphate, pH 7.5, from the isolation buffer and concentrated. Cytochrome was added to a quartz cuvette, along with a 2 × 7 mm stir bar. The cuvette was transferred into the glovebox where the cytochrome was diluted to 2.9 mL with previously degassed 100 mM potassium phosphate, pH 7.5. to achieve a final concentration of 15 μM wild-type (WT) PpcA and 2.5 μM K29C-Ru and K52C-Ru. A solution of redox mediators consisting of 1,4-naphthoquinone, anthraquinone, 2-hydroxy-1,4-naphthoquinone, and duroquinone (all from Sigma-Aldrich, St. Louis, MO, USA) in ethanol was also added. The cytochrome solution was then degassed using gentle argon bubbling for 45 min to remove any residual dissolved oxygen. After degassing, the cytochrome was reduced using 100 μL of 40 mg/mL sodium dithionite prepared in degassed 100 mM potassium phosphate, pH 7.5, and was allowed to equilibrate for 10 min until the voltage was stable at −300 to −350 mV. The cytochrome was then titrated using 650 μM potassium ferricyanide in degassed 100 mM potassium phosphate, pH 7.5, at a rate of 5 μL/hour. Titrations were performed for 16 h or until the voltage had stabilized with less than 5 mV of change per hour. Potentials were recorded with a freshly cleaned Pt wire and an Ag/AgCl reference electrode. Optical spectra were continuously recorded with a Thor Labs CCS200 spectrophotometer. 

All-atom molecular dynamics (MD) simulations were performed using computational resources of the Laboratory Computing Resource Center (Argonne National Laboratory, Lemont, IL, USA). Initial atom coordinates were taken from the first frame of 2LDO NMR structure of PpcA. In silico mutations were introduced using VMD [58] and the system with an explicit water box was allowed to equilibrate for 10 ns in all-atom molecular dynamics simulations performed with NAMD2 [59]. This step was followed by addition of covalently linked Ru(II)(bpy)_2_(Cys-bpy) photosensitizers and simulations were continued with the parameters as described previously [51]. For all labeled biohybrids and wild-type PpcA, three independent ~300–400 ns long simulation were performed. The intermediate structural snapshots were recorded each 10 ps and were analyzed with VMD. 

Time-resolved transient absorbance measurements were performed using a Ti:Sapphire pumped optical parametric amplifier (OPA) with 120-fs pulsewidth and tuned to 460 nm for excitation of the Ru(II)(bpy)_3_ photosensitizer at the Nanophotonics User Facility of the Center for Nanomaterials, Argonne National Laboratory (Lemont, IL, USA) with experimental settings as previously described [15]. We did not investigate the dependence of kinetics on the excitation wavelength; 460 nm corresponds to the absorbance maximum of Ru(bpy)_3_. Because of the significantly higher extinction coefficients of the hemes at other wavelengths, there are no practical spectral regions for selective photoexcitation of Ru(bpy)_3_ outside of 460 nm. To obtain kinetic reaction parameters, we used least squares fitting with bi-exponential functions in Origin using the difference between the absorbance values at 553 and 541 nm. In our previous work [51] we have demonstrated that the time constant for the rising edge of PpcA reduction for K29C-Ru matched the time constant of the decay of Ru(II*)(bpy)_3_ and assigned it to the charge separation (CS) process. Since we expect the free energies of charge separation and recombination to be similar for the other three constructs, we similarly interpret that the rising edges of these PpcA reduction kinetics are also due to CS. The falling edge is correlated with recovery of the ground state spectra and we interpret this process as charge recombination (CR). Kinetic models to describe TA kinetics in Ru-labeled multi-heme cytochromes include those which consider a multi-state scheme, and are found to favor a kinetic scheme where CR is faster than CS [60], and those which assume a simple two-state kinetic model, where CS is faster than CR [51,52]. Unfortunately, the present TA data do not provide a detectable signature of the CR kinetics for Ru(III)(bpy)_3_, so we do not have a direct, independent way to detect the CR reaction and distinguish these two kinetic models. For the moment, we prefer the two-state kinetic model in which CS is assumed to be faster than CR for two reasons. First, because this kinetic model was found to yield a fit to the free energy dependence for the TA kinetics for the Ru-labeled PpcA constructs using the Marcus equation and with fitting parameters that are physically realistic and consistent to those in other biological-base electron transfer systems [51]. Second, because the multistate model when applied to Ru-labeled cytochromes, where the TA features rise on a few ps timescale, would result in requiring faster CR rates to be potentially unrealistically fast, on the fs timescale. Further work is on-going to distinguish between the applications of these two kinetic models.

Small- and wide-angle X-ray scattering (SAXS and WAXS, respectively) experiments were performed at the 12-ID-B beamline of the Advanced Photon Source at Argonne National Laboratory (Lemont, IL, USA). Protein samples in 20 mM Tris buffer, pH 7.5 with 100 mM NaCl were concentrated to yield final protein concentration in the range of 0.2–0.5 mM. To minimize protein damage by X-ray radiation, samples were slowly refreshed in a flow cell with a syringe pump. All other parameters of combined SAXS/WAXS data collection were the same as described previously [56]. 

## 3. Results

### 3.1. Protein Expression, Purifiction and Photosensitizer Labeling

The mutant forms of PpcA were expressed in *E. coli* as previously described [51,52]. We obtained protein yields comparable to wild-type PpcA. LC-MS confirmed the purity of our isolated proteins and the observed masses matched the calculated masses based on the DNA sequence and covalent attachment of 3 c-type hemes. Similar to the findings in our previous work, in the absence of DTT or other thiol reductants we observed increased protein masses and substantial heterogeneity of the eluted PpcA fractions. However, protein isolations prepared with all washing and elution buffers supplemented with 1 mM DTT resulted in one dominant PpcA fraction and protein masses consistent with monomeric protein without any post-translational modifications. This observation suggests that the engineered cysteine residues are reversibly post-translationally modified with *E. coli* metabolites.

Covalent modification of the mutant forms of PpcA with Ru(II)(bpy)_2_(Br-bpy), the photosensitizer selected for this work, did not produce any unexpected results. Because of a five-fold molar excess of photosensitizer over protein and the substantially different masses of the protein-photosensitizer hybrids and the sensitizer alone, a simple and quick separation on a size exclusion Sephadex G25 column was sufficient to obtained > 95% covalently labeled protein based on LC-MS characterization. The observed and expected protein masses are shown in Table S2.

To verify correct protein folding after genetic mutation and chemical attachment of a charged and bulky photosensitizer molecule to PpcA, we performed synchrotron small-angle X-ray scattering characterization of wild-type PpcA and all four covalent constructs. Guinier plots prepared after subtraction of solvent contribution to the X-ray scattering are shown on Figure S1. All five samples show straight line dependencies with similar slopes. The calculated radii of gyration (R_g_) are summarized in Table S3. The similarity of sizes indicates that mutations and covalent photosensitizer attachment to the biohybrids do not affect the compact globular structure of proteins. A slight increase in R_g_ values for K28C-Ru and G53C-Ru and increased slopes at low q values further suggest that these two complexes have an elongated form likely due to protrusion of the photosensitizers from the protein surface. 

To further verify structural stability of PpcA biohybrids and to test for preferential binding of Ru(II)(bpy)_2_(Br-bpy) enantiomers, we collected circular dichroism (CD) spectra at temperatures between 25 °C and 90 °C. Consistent with our recent report [52] that showed preferential binding of the Δ enantiomer of Ru(II)(bpy)_3_ to K29C and Λ enantiomer to A23C, we observed a strong preference for binding of the Λ enantiomer by K52C, and a weak preference for the Δ enantiomer by K28C (Figure S2). Similar to the previously reported E39C-Ru biohybrid, we did not observe a significant enantiomer selection with G53C. K29C-Ru biohybrid appears to be slightly more stable to thermal denaturation than WT PpcA. For the other three labeled mutants we did not observe any significant deviation from the melting curve of the wild-type form (Figure S3). These results suggest that cysteine mutations and covalent labeling with relatively big and charged Ru(II)(bpy)_3_ did not destabilize protein structure for these four mutants of PpcA.

Extensive prior work from the Salgueiro group has demonstrated that surface charge modifying mutations in PpcA can cause noticeable changes in the heme midpoint potentials [61,62,63,64,65]. We anticipated that biohybrid constructs reported in this work may also have perturbed heme potentials since: (a) three out of four constructs had positive lysine residues replaced with neutral cysteines; (b) the covalently attached photosensitizers have a +2 charge, (c) mutations and attachment of bulky photosensitizer molecules are likely to change solvent exposure of heme edges. For K52C-Ru and K29C-Ru we observed +39 mV and +31 mV increases in apparent midpoint potentials in comparison with wild-type PpcA (Figure S4). The magnitude of the changes is comparable to the most significant changes for acidic-to-basic mutants reported by the Salgueiro group. Because of the anticipated longer distance between hemes and photosensitizer in K28C-Ru and G53C-Ru, we expect the midpoint potentials of these constructs to fall between the values observed in the wild-type form and K29C-Ru and K52C-Ru constructs.

### 3.2. Kinetics of Electron Transfer

**Figure 2:**
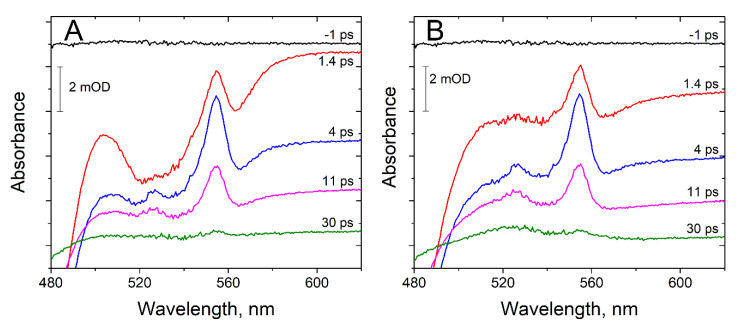
Spectral changes in K52C-Ru biohybrid at selected time delays in pump-probe transient absorbance experiments. (**A**) chirp-subtracted differential spectral changes vs. equilibrium ~1 ms after the pump pulses. Time delays are shown on the right of each spectrum. The spectra are vertically offset for clarity. (**B**):transient absorbance spectra with contributions from excited state hemes subtracted.

**Figure 3:**
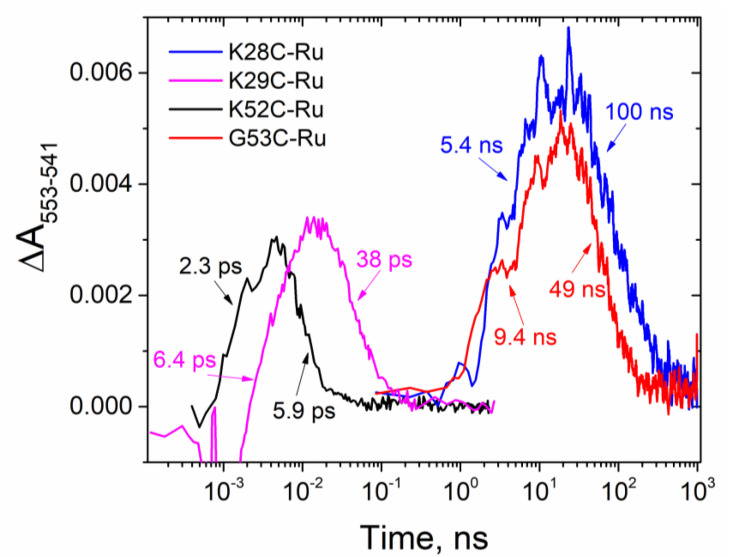
Kinetics of photo-induced electron transfer in biohybrids.

Pump-probe transient absorbance spectra collected 1 ps before the excitation pulses show no spectral features (Figure 2A) and are indistinguishable from solvent response data (not shown) collected with the same timing. This suggests that our biohybrid constructs do not have any populations with lifetimes comparable to or slower than half of the time period between the pump pulses (1k Hz repetition rate, each second pump pulse blocked from reaching the sample cuvette). However, spectra recorded shortly after pump pulses show complex kinetics and multiple sub-populations (Figure 2A). The kinetic is particularly complex for K29C-Ru and K52C-Ru and to quantitatively evaluate the picosecond kinetics of heme reduction observed near 553 nm, we corrected for the chirp and solvent response and also subtracted the heme-excited state dynamics. The resulting corrected spectra (Figure 2B) show the characteristic sharp band for the reduction of c-type hemes near 553 nm and also a much broader band with maximum near 650 nm assigned to the photosensitizer excited state (Ru(II)(bpy)_3_*) and Ru(III)(bpy)_3_. In contrast, no spectral corrections were needed for K28C-Ru and G53C-Ru (Figure S5) as they did not show any sub-nanosecond kinetics. Beyond the first 20 ps after the pump pulse, the spectral contributions from chirp, solvent response, and heme excited state dynamics were negligible. Figure 3 shows the kinetics of ET for all four PpcA-Ru(II)(bpy)_3_ biohybrids, and summarizes the apparent time constants for CS and CR.

Contrary to our expectations of ultrafast charge transfer for all attachment sites within the heme binding domains, we observed ultrafast apparent charge separation only in previously reported K29C-Ru (6.4 ± 0.4 ps) and the new construct K52C-Ru (2.3 ± 0.2 ps) (Figure 3). The latter is the fastest apparent photo-induced charge separated time constant for hybrid protein-photosensitizer complexes reported to date and reaches the values observed in natural photosynthesis. In sharp contrast, the photosensitizer attachment to K28C-Ru and G53C-Ru yielded ET kinetics of about three orders of magnitude slower, with the time constants of 5.4 ± 0.5 ns and 9.4 ± 0.8 ns, respectively (Table S4). We observed a variation of apparent ratios of charge transfer time constants: 2.6-fold for K52C-Ru, 5.2-fold for G53C-Ru, 5.9-fold for K29C-Ru, and 18.5-fold for K28C-Ru. Since all three heme redox potentials are similar, we expected the ratios of charge separation to charge recombination rates to be fairly close to each other for all four constructs. A notably slower relative charge recombination in K28C-Ru may indicate stabilization of the charge-separated state through electron transfer from the proximal to Ru(II)(bpy)_3_ heme to one of the distal cytochrome hemes similar to the effect reported by van Wonderen and co-workers [60].

### 3.3. Molecular Dynamics Modeling

**Figure 4:**
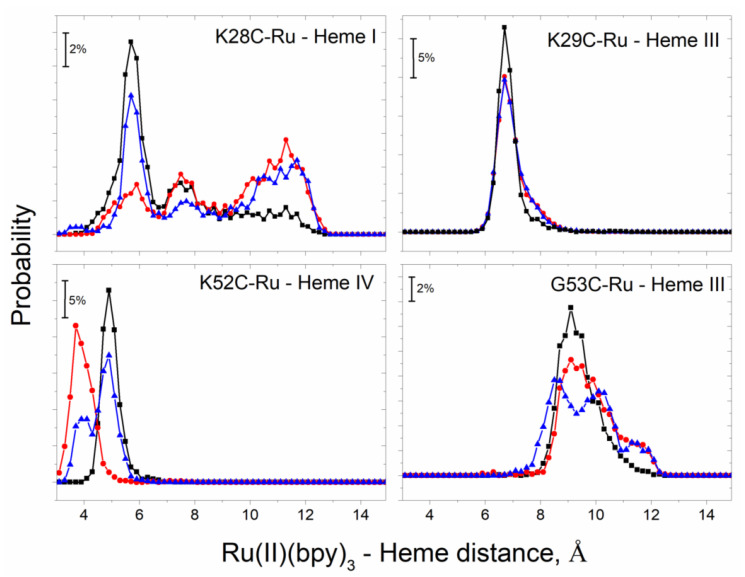
Distance distributions between aromatic atoms of photosensitizer and the closest PpcA hemes as observed in three independent (shown with different colors) ~300 ns each all-atom molecular dynamics simulations (each simulation shown with different colors). Frequencies (in %) were calculated for 0.2 Å distance intervals.

To gain a better understanding of the structural requirements for ultrafast ET in protein-photosensitizer hybrids, we performed all-atom molecular dynamics simulations with explicit solvent in triplicate. It was previously demonstrated that the shortest distance between aromatic atoms of donors and acceptors can serve as a useful predictor of the experimental ET rates [36,37]. Figure 4 shows the distributions of minimal DA aromatic atom distances for all four constructs. For K28C-Ru (Figure 4) we observed broad distributions for all three simulations. In contrast, for K29C-Ru all three distance distributions formed narrow peaks with maxima near 6.6 Å. Another construct with ultrafast photo-induced electron transfer (PET) kinetics, K52C-Ru (Figure 4) showed two subpopulations with narrow distributions of distances: one conformation with 3.8 Å distance and the other with 4.9 Å. Since we observed clear monoexponential charge separation and decay, it is likely that one of the observed sub-populations is an artifact of the inherent time scale limitations of all-atom molecular dynamics simulations and challenges associated with obtaining reliable weighting of local minima populations. Finally, for G53C-Ru we observed qualitatively different results from the three other constructs: not only the average distances were substantially longer (~9.5 Å) but also the widths of those distributions were noticeably broader. This suggests a mobile photosensitizer without significant steric constraints from the protein structure and consistent with the absence of stereoisomer selection.

**Figure 5:**
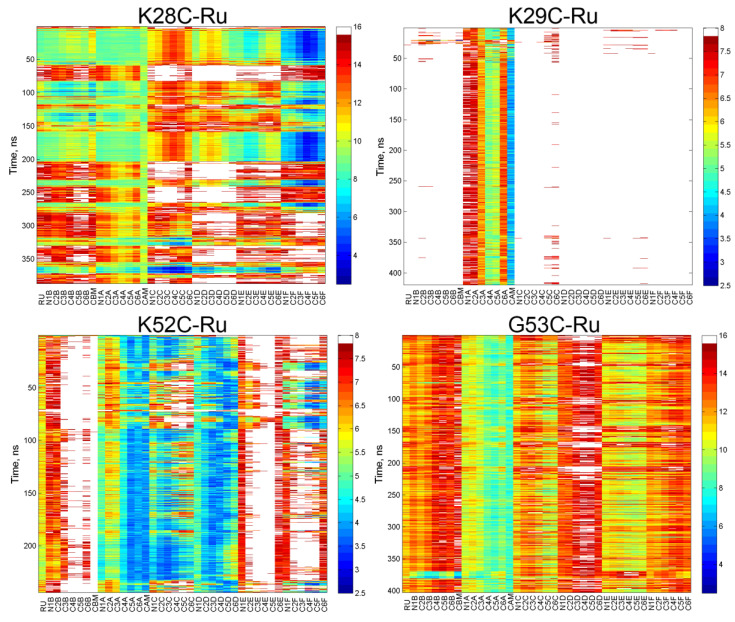
Shortest distances between photosensitizer atoms (*x*-axes) and closest aromatic atoms of PpcA hemes from representative MD simulations (time on *y*-axes) with distances coded according to the color bars on the right of each panel.

**Figure 6:**
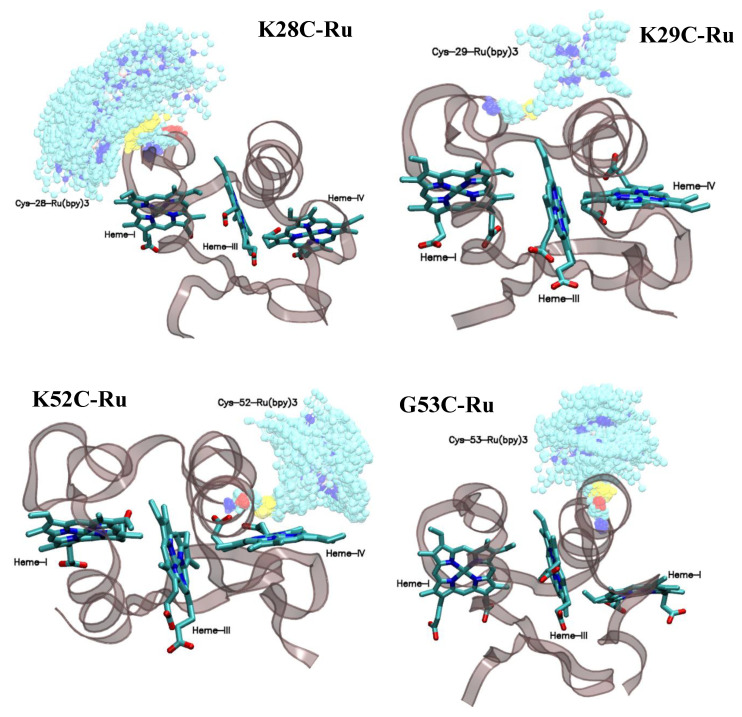
PpcA structures with overlaid photosensitizer structures from ~3 ns separated in time MD snapshots.

To further explore molecular level requirements for ultrafast ET in biohybrids, we compared the shortest individual distances between all aromatic atoms of Ru(II)(bpy)_3_ to the closest aromatic heme atoms (Figure 5). In K28C-Ru simulations (Figure 5) we initially observed ~4 Å distances between carbon atoms of one of the bipyridines and aromatic atoms of the closest heme. However, that conformation was not stable and showed changes on the time scale of tens of nanoseconds and moved to a position with minimal DA distance of about 10 Å after ~250 ns. If correct, this would indicate that initial short photosensitizer–heme distances are an artifact of in silico mutations/labeling and consistent with the absence of ultrafast kinetic components expected from van der Waals contacts between the DA pair. Similarly, the absence of a preferential Ru(II)(bpy)_3_ stereoisomer selection for this construct is consistent with a mobile photosensitizer bound on the surface of the protein (Figure 6).

While the observed average photosensitizer-heme distance of about 6.6 Å in K29C-Ru is still shorter than that in most protein-photosensitizer constructs studied by the Gray [19,20,21,22,28,31,34] and Millett [24,25,26,29] groups, it is surprising that this distance significantly exceeds the van der Waals contact distance and still can facilitate ET with a 6.4 ps time constant. Another notable feature is the very narrow and reproducible distributions of distances for this construct. The observed MD results are consistent with the significant Ru(II)(bpy)_3_ enantiomer selection recently reported by our group [51] and are a highly sterically restricted binding site (Figure 6).

From K52C-Ru simulations it appears that the sub-population with ~3.9 Å DA distance is present only at equilibration and is likely to be an artifact (Figure 5). The other sub-population has somewhat longer average distances, but at 4.9 Å it is substantially shorter than any previously reported construct. While it seems to be more flexible and disordered (Figure 5 and Figure 6) than K29C-Ru, one of the bipyridine rings moves along the Heme-IV plane and maintains a very short DA distance. 

Finally, for G53C-Ru the simulations converge in about 200 ns to configurations with average DA distances of ~8 Å with substantial mobility and Ru(II)(bpy)_3_ noticeably protruding away from the protein surface (Figure 6). The observed mobility is consistent with the absence of enantiomer selection in the circular dichroism experiments. The protrusion of the photosensitizer molecule away from the protein surface is consistent with our SAXS data and Guinier plots predicting an elongated G53C-Ru solution-state structure. 

## 4. Discussion

In this report we have characterized ET kinetics for a series of photosensitizer-labeled PpcA constructs, K28C-Ru, K52C-Ru, and G53C-Ru. Together with K29C-Ru reported previously [51], this series provides a comparison of ET initiated from a Ru(II)(bpy)_3_ photosensitizer linked to comparable sites within the heme binding motif sequences (CXXCH) for two different cofactor sites, Heme-I and Heme-III, in the multiheme c-cytochrome, PpcA. Each site shares a comparable through-bond pathway to a covalently linked heme. The results show a substantial variation in ET kinetics spanning a range over three orders of magnitude, with K52C-Ru showing charge transfer and recombination kinetics of 2.3 ps and 5.9 ps, respectively, while G53C-Ru showed the slowest charge transfer and recombination kinetics of 9.4 ns and 49 ns, respectively. The results demonstrate that ultrafast ET can be designed in PpcA as a charge-accepting framework, but that tertiary structure and the strength of electronic coupling between photosensitizer and heme cofactors are critically varying, site-specific factors. 

The targeting of variable residue sites within the heme attachment CXXCH consensus sequences for mutagenesis and covalent linkage of photosensitizers provides an opportunity to investigate ET dynamics with a series of constructs that maintain constant through-bond pathways, but have varying through-space pathways and non-covalent contacts between the attached photosensitizer and heme cofactors. However, mutations that convert variable residues to cysteines next to universally conserved cysteine residues functioning as heme attachments pose risks of having the engineered cysteines disrupt heme attachment or protein folding during c-type cytochrome maturation. That cysteine mutation at the CXXCH variable sites does not disrupt PpcA maturation is supported by several methods of structural characterization. 

LC-MS results shown above unambiguously demonstrate the covalent attachment of all three hemes of PpcA, which is followed by successful chemical modification of the engineered cysteine residues with the cysteine-reactive Ru(II)(bpy)_3_-derivative photosensitizer. X-ray scattering measurements demonstrate that the proteins with cysteine mutations retain their native globular shape even after photosensitizer attachment. Slight deviations from linearity at low momentum transfer vector values on Figure S1 observed for K28C-Ru and G53C-Ru are consistent with a flexible linkage having the photosensitizers protruding from the protein surface as observed by MD simulations (Figure 6). In contrast, compact globular forms of K29C-Ru and K52C-Ru inferred from the Guinier X-ray scattering plots (Figure S1) are consistent with sterically restricted sites observed in MD simulations (Figure 6) and steric hindrances resulting in Ru(II)(bpy)_3_ enantiomer selection (Figure S2), and as reported previously for K29C-Ru [52]. We note that K29C-Ru and K52C-Ru show a strong selectivity for enantiomers of the opposite chirality for the Ru(II)(bpy)_3_ derivative, demonstrating that the chiral selectivity arises not from the covalent linkage, but from the stereo-specificity of the binding site. In addition, the observation of ultrafast ET characteristics for K29C-Ru and K52C-Ru support the contention that chiral selectivity for the Ru(II)(bpy)_3_ derivative is a useful marker for a tight, conformationally-selective structure, which is a necessary condition to achieve uniform, ultrafast ET [52]. Finally, the temperature dependence of circular dichroism signal recorded at 222 nm and corresponding to the ordered secondary protein structure is indistinguishable for all four constructs from the wild-type form of PpcA at least up to 90 °C. Collectively, these results suggest that PpcA retains its globular shape despite mutations and covalent labeling. 

Transient absorbance results for K52C-Ru revealed an even faster charge separation and recombination kinetics than previously reported by our groups for K29C-Ru [51] and slightly faster than the fastest component recently reported for a similar multiheme cyt c-Ru(II)(bpy)_3_ construct based on cytochrome STC from *Shewanella oneidensis* with very short heme-photosensitizer (~5.0 Å) distances [60]. In the absence of reliable experimental structural data, we rely on MD simulations to predict DA distances. Assuming that the distances are accurate, we have a growing number of examples illustrating that the shortest distances between aromatic atoms cannot explain the precise ordering of observed ultrafast ET rates, although each features short range minimal distances (<7 Å). This indicates that a simple minimal distance in MD simulations between aromatic atoms of DA pairs may not be a reliable predictor of specific ultrafast ET rates, and that a more complete analysis of electronic coupling and orbital overlap integrals between donor and acceptor moieties is needed. Similarly, more than 1000-fold slower ET rates for K28C-Ru and G53C-Ru rates suggest that contrary to our hypothesis outlined above, the lowest possible number of covalent bonds between donor and acceptor molecules in c-type cytochromes do not serve as a reliable predictor of ET rates either. Interestingly, all three ultrafast PpcA constructs (A23C-Ru, K29C-Ru, K52C-Ru) reported in our publications (this work and [51,52]) show significant Ru(II)(bpy)_3_ enantiomer selection during binding and sterically restricted attachment sites. K29C and K52C show higher enantiomer selectivity than A23C. This result implies more specific and tighter binding sites for K29C and K52C and correlates with substantially faster ET rates for these two mutants in comparison with A23C. Considering that the excited state energy of the photosensitizer (~2.0 eV) is almost two orders of magnitude greater than the energy of thermal fluctuations (~25 meV), even vibrational cooling of the photosensitizer to its lower excited state levels is likely to produce significant local heating on the ultrafast time scale [66,67,68,69,70,71,72,73,74,75,76,77,78,79,80,81,82,83,84,85,86,87,88]. This effect should significantly perturb the equilibrium DA distances and coupling, resulting in much slower ET in the constructs with mobile photosensitizers. By contrast, the biohybrids with immobile photosensitizers tightly coupled to protein frameworks should be able to dissipate heat energy more effectively and to better maintain donor-acceptor coupling resulting in faster ET. This is clearly visible in primary charge separated events in various natural photosynthetic systems, but for the first time is demonstrated in an artificial system with the surface-bound photosensitizers [51,52]. 

The observed 1000-fold difference in the observed ET rates between the ultrafast K29C-Ru and K52C-Ru constructs and much slower K28C-Ru and G53C-Ru cannot be explained by the difference in the free energies of the reactions due to more distant photosensitizers affecting the heme redox potentials less than if they were positioned more closely. Based on the Marcus model, previously reported reorganization energy λ = 0.85 eV [51] for PpcA-Ru, and driving forces located near the top of the Marcus parabola, the 40 mV variation in heme potential induced by mutations and covalent labeling will have only a minor effect on the ET rates (Figure S6). A much more significant change of about 600 mV is needed to achieve 1000-fold slow-down of the ET rates that we observe here. 

Marcus theory dictates that approximately the same driving forces for charge separation and recombination steps should result in similar ratios of charge separation and recombination rates. We observed this effect for the five previously reported mutants [51]. This trend is followed by three out of the four constructs reported in this work. However, for K28C-Ru the ratio of the apparent charge recombination to charge separation is about 18, significantly more than 3–6-fold range observed for the other eight biohybrid PpcA-Ru constructs in this and our previous work [51]. Considering that for K28C-Ru, the Ru(II)(bpy)_3_ photosensitizer is expected to be in this construct near Heme-I with the lowest redox potential of all three hemes [89,90], one possibility for the significant slow-down of charge recombination for this construct can be due to rapid electron transfer to higher potential distal hemes of PpcA and stabilization of the charge-separated state similar to electron transfer chains of natural photosynthesis. Although the rate of heme-heme ET for PpcA was never measured before, this observation also sets the time scales of intra-protein ET transfer in PpcA much faster than 5 ns. If this were not the case, we would have observed a multi-phase charge recombination kinetics similar to those reported by van Wonderen and co-workers in a tetraheme cytochrome [60]. 

The work presented here demonstrates the opportunity to design ultrafast, light-driven charge injection into multi-heme c-type cytochrome “nanowires” by targeting variable residue sites within CXXCH heme attachment. The results show that the ET mechanism proceeds by direct, through-space, photosensitizer-heme cofactor contacts rather than through-bond pathways. MD simulations are shown to provide a qualitative rather the quantitative predictor of biohybrid conformations and mutation sites that support ultrafast ET. This work demonstrates opportunities to utilize multi-heme c-cytochromes as frameworks for designing ultrafast light-driven ET into charge-accumulating biohybrid model systems, and ultimately for mimicking the photosynthetic paradigm of efficiently coupling ultrafast, light-driven ET chemistry to multi-step catalysis within small, experimentally versatile photosynthetic biohybrid assemblies.

## Supplementary Materials

The following are available online at https://www.mdpi.com/2079-4991/10/11/2143/s1, Table S1: DNA oligonucleotides used to produce K28C, K52C, and G53 mutations in PpcA; Table S2: Observed protein masses (in Da) with electrospray ionization mass spectrometry (ESI-MS); Figure S1: Guinier plots of wild-type PpcA and covalently labeled PpcA biohybrids; Table S3: Radii of gyration (in Å) of wild-type PpcA and Ru(II)(bpy)_3_ -labeled mutants; Figure S2: Circular dichroism spectra for cysteine-labeled mutants vs. wild-type PpcA; Figure S3: Normalized ellipticity at 222 nm over the range of 25–90°C; Figure S4: Redox titrations reveal that mutations and covalent labeling with Ru(II)(bpy)_3_ result in relatively minor increase of the apparent midpoint potential of cytochrome biohybrids; Figure S5: Spectral changes in K28C-Ru, K29C-Ru, and G53C-Ru biohybrids at selected time delays in pump-probe transient absorbance experiments; Table S4: Time constants for charge separation and recombination for biohybrids; Figure S6: Expected change in the rate of charge separation based on the shift of heme redox potential calculated from the Marcus model.
