# Supplementary material for: Mimicking Natural Photosynthesis: Designing Ultrafast Photosensitized Electron Transfer into Multiheme Cytochrome Protein Nanowires
Source: Nanomaterials (Basel). 2020 Oct 28;10(11):2143. doi: 10.3390/nano10112143 (PMC7693585; doi:10.3390/nano10112143)
Supplement: Supplementary file 1 [file nanomaterials-10-02143-s001.pdf]

nanomaterials

Supplementary Materials

Mimicking Natural Photosynthesis: Designing Ultrafast Photosensitized Electron Transfer into Multiheme Cytochrome Protein Nanowires

Daniel R. Marzolf [1,†], Aidan M. McKenzie [1,‡], Matthew C. O'Malley [1,§], Nina S. Ponomarenko [2], Coleman M. Swaim [1,‖], Tyler J. Brittain [1], Natalie L. Simmons [3], Phani Raj Pokkuluri [4,¶], Karen L. Mulfort [2], David M. Tiede [2] and Oleksandr Kokhan [1,*]

1 Department of Chemistry and Biochemistry, James Madison University, Harrisonburg, VA 22807, USA; marzolf.4@buckeyemail.osu.edu (D.R.M.); mckenzie4@wisc.edu (A.M.M.); matthew.o'malley@ppdi.com (M.C.O.); coleman.swaim@quinnipiac.edu (C.M.S.); brittatj@dukes.jmu.edu (T.J.B.)
2 Chemical Sciences and Engineering Division, Argonne National Laboratory, Lemont, IL 60439, USA; ponomarenko@anl.gov (N.S.P.); mulfort@anl.gov (K.L.M.); tiede@anl.gov (D.M.T.)
3 Department of Biology, James Madison University, Harrisonburg, VA 22807, USA; simmo2nl@dukes.jmu.edu
4 Biosciences Division, Argonne National laboratory, Lemont, IL 60439, USA; prp0015@auburn.edu
* Correspondence: kokhanox@jmu.edu; Tel.: +1-540-568-1656
† Present address: Biophysics Program, The Ohio State University, Columbus, OH 43210, USA.
‡ Integrated Program in Biochemistry, University of Wisconsin-Madison, Madison, WI 53706, USA.
§ PPD Inc., Richmond, VA 23230, USA.
‖ F.N. Netter School of Medicine, Quinnipiac University, Hamden, CT 06518, USA.
¶ Department of Chemistry & Biochemistry, Auburn University, Auburn, AL 36849, USA.

Supplementary Materials

Name	Sequence
K28Cfor	TCCCGACTGTtgcAAGTGCCACGAG
K28Crev	ACAGCCTTCTGGTGGGCC
K52Cfor	CAAGGGCTGCtgcGGGTGCCACG
K52Crev	CCATGAGCCATCTCTTTGC
G53Cfor	GGGCTGCAAGtgcTGCCACGAAG
G53Crev	TTGCCATGAGCCATCTCTTTG

Table 1. DNA oligonucleotides used to produce K28C, K52C, and G53 mutations in PpcA. K29C was available from our previous work [1,2].

Name	Observed mass, Da	Expected mass, Da
Wild-type	9,583	9,583
K28C-Ru	10,150	10,151
K29C-Ru	10,151	10,151
K52C-Ru	10,151	10,151
G53C-Ru	10,222	10,222

Table 2. Observed protein masses (in Da) with ESI-MS.

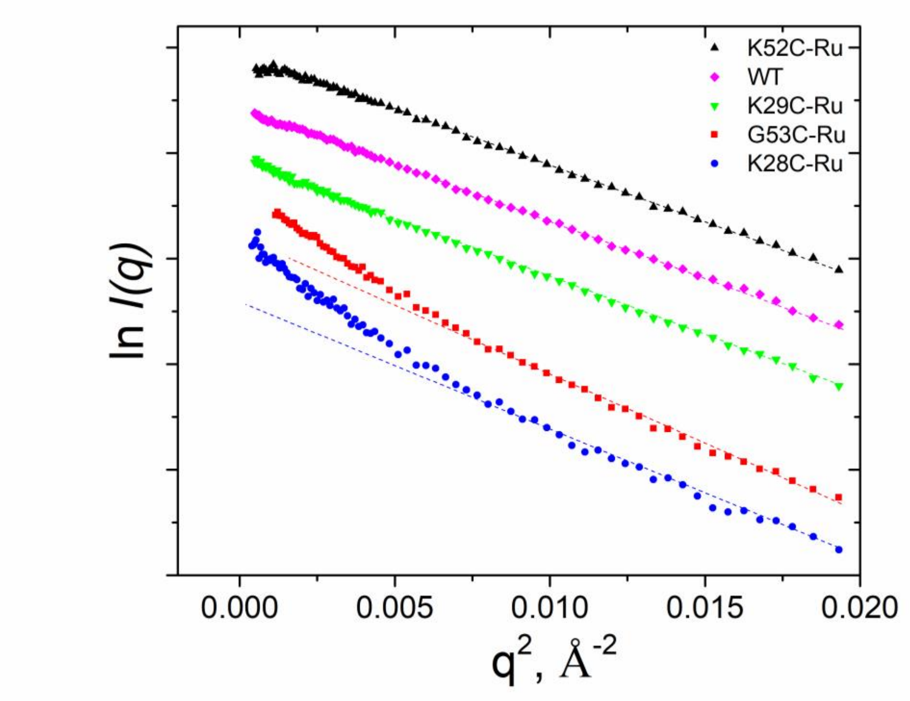

Figure 1. Guinier plots of wild-type PpcA (♦) and covalently labeled PpcA biohybrids: K28C-Ru (●), K29C-Ru (▼), K52C-Ru (▲), and G53C-Ru (■).

Name	Gyration radius, Å
Wild-type	12.6 ± 0.2
K28C-Ru	14.0 ± 0.3
K29C-Ru	12.8 ± 0.2
K52C-Ru	12.6 ± 0.2
G53C-Ru	14.3 ± 0.3

Table 3. Radii of gyration (in Å) of wild-type PpcA and Ru(II)(bpy)$_3$ -labeled mutants.

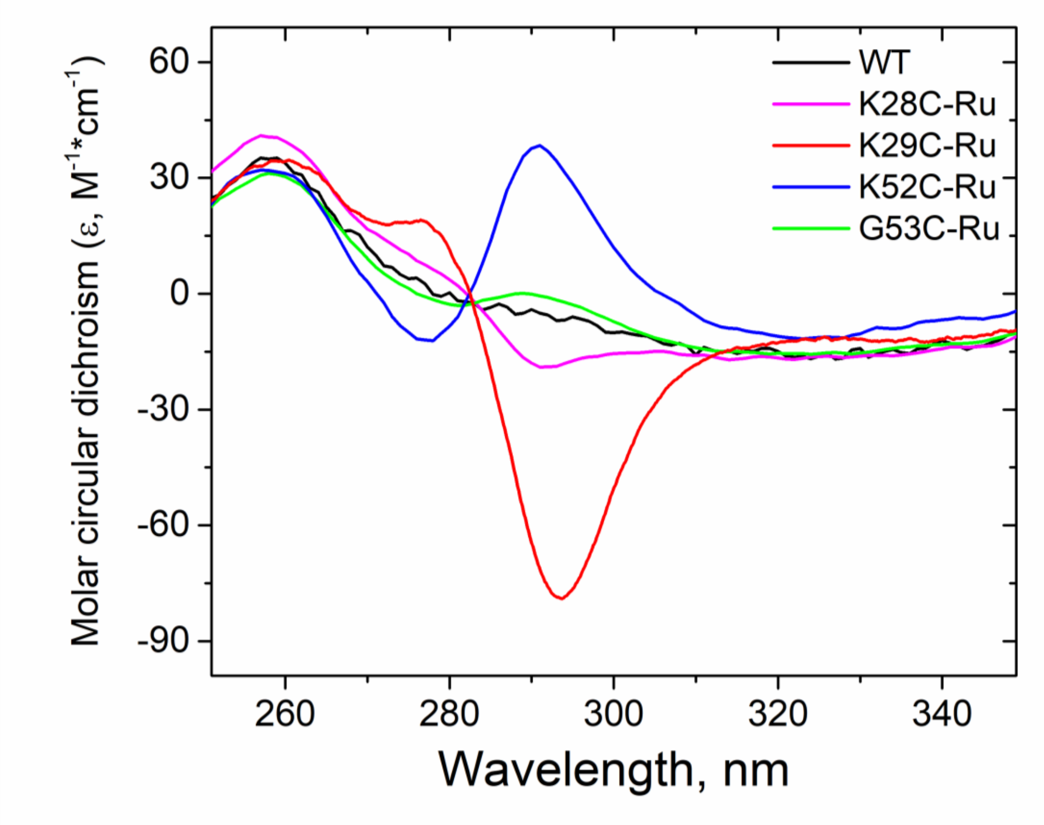

Figure 2. Circular dichroism spectra for cysteine-labeled mutants vs. wild-type PpcA reveal strong Ru(II)(bpy)3 enantiomer selection for K29C-Ru and K52C-Ru.

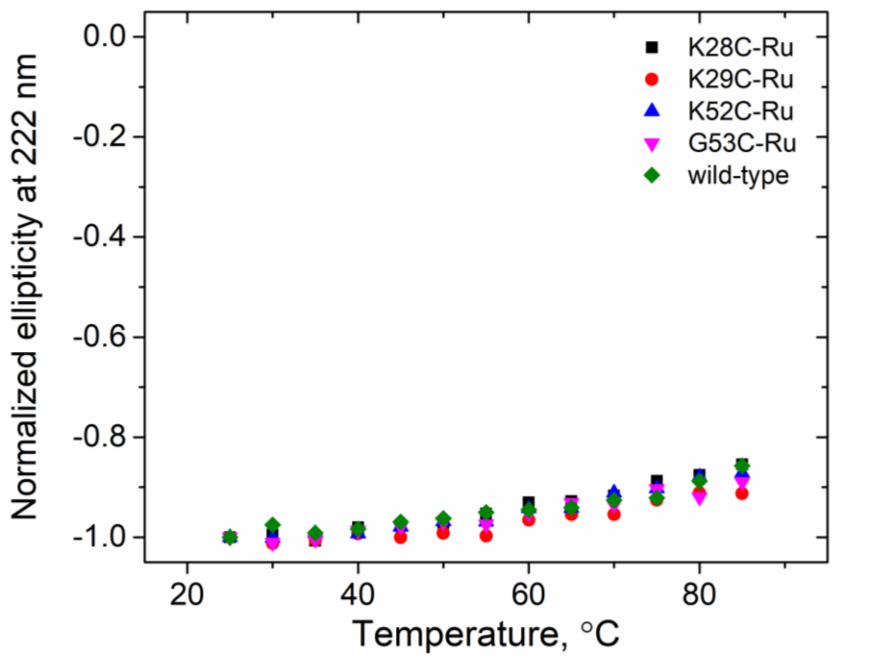

Figure 3. Normalized ellipticity at 222 nm over the range of 25-90°C demonstrates that mutations and covalent labeling of the mutants result in biohybrids resistant to thermal denaturation over this temperature range and show thermal stability comparable to the wild-type protein form.

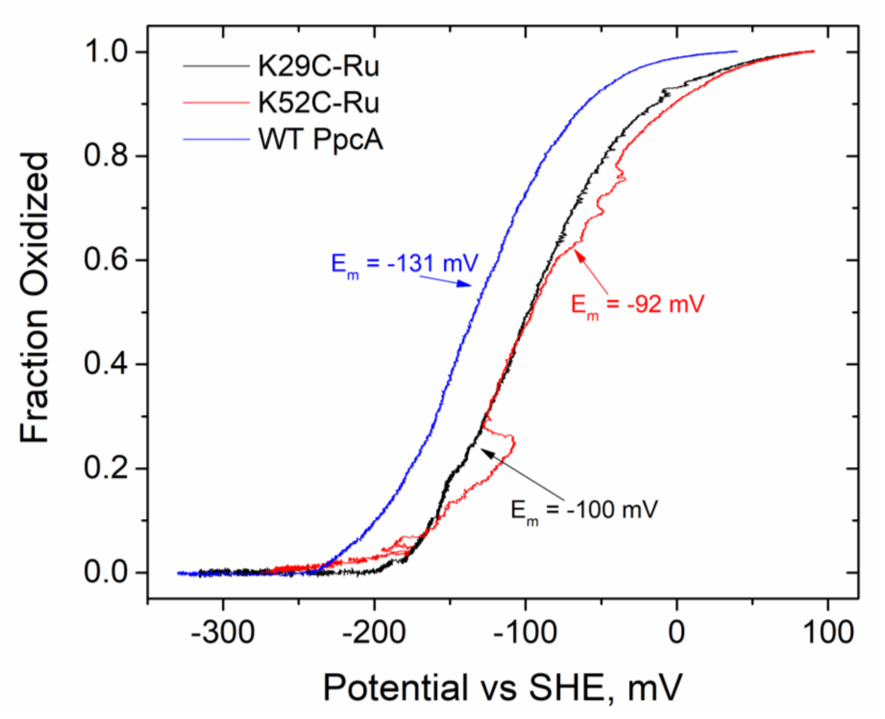

Figure 4. Redox titrations reveal that mutations and covalent labeling with Ru(II)(bpy)$_3$ result in relatively minor increase of the apparent midpoint potential of cytochrome biohybrids. Corresponding midpoint potential from non-linear curve fitting are shown on the graph.

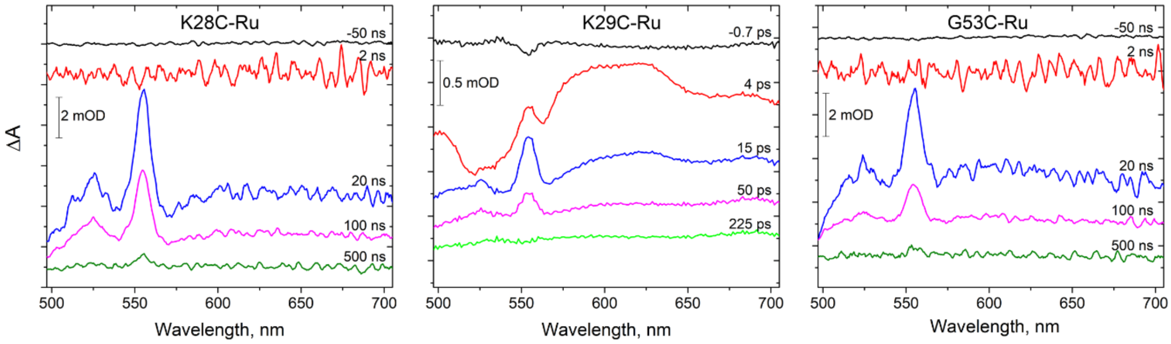

Figure 5. Spectral changes in K28C-Ru (**left**), K29C-Ru (**center**), and G53C-Ru (**right**) biohybrids at selected time delays in pump-probe transient absorbance experiments.

Name	Time of charge separation	Time of charge recombination
K28C-Ru	5.4 ± 0.5 ns	100 ± 5 ns
**K29C-Ru**	**6.4 ± 0.4 ps**	**38 ± 2 ps**
**K52C-Ru**	**2.3 ± 0.2 ps**	**5.9 ± 0.4 ps**
G53C-Ru	9.4 ± 0.8 ns	49 ± 3 ns

Table 4. Time constants for charge separation and recombination for biohybrids. Ultrafast rates are bolded for clarity.

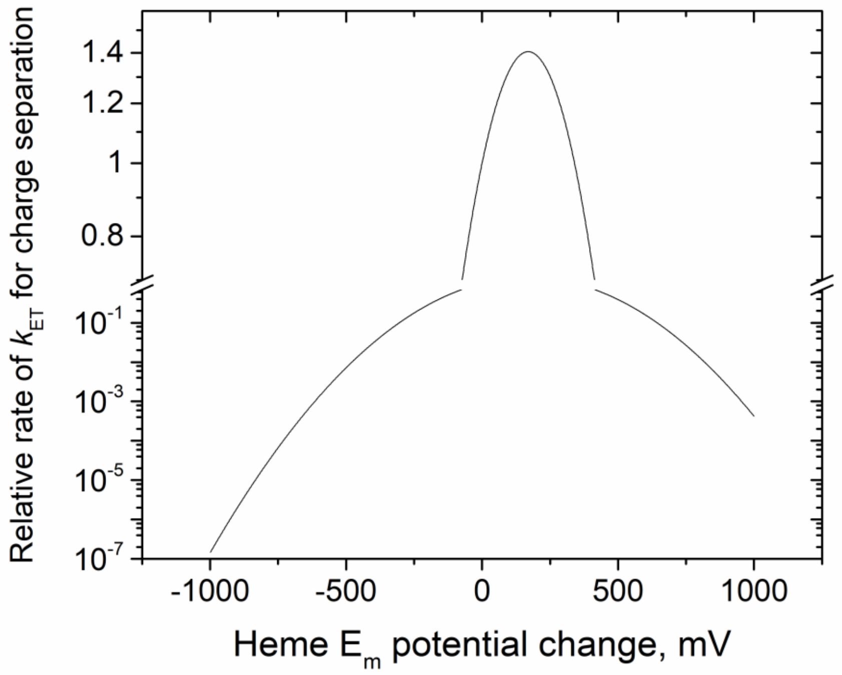

Figure 6. Expected change in the rate of charge separation based on the shift of heme redox potential calculated from the Marcus model assuming reorganization energy λ=0.85 eV [1] and ΔG=0.72eV.

References

1. Kokhan, O.; Ponomarenko, N.S.; Pokkuluri, P.R.; Schiffer, M.; Mulfort, K.L.; Tiede, D.M. Bidirectional Photoinduced Electron Transfer in Ruthenium(II)-Tris-bipyridyl-Modified PpcA, a Multi-heme c-Type Cytochrome from Geobacter sulfurreducens. *J Phys Chem B* **2015**, *119*, 7612-7624, doi:10.1021/jp511558f.
2. Ponomarenko, N.S.; Kokhan, O.; Pokkuluri, P.R.; Mulfort, K.L.; Tiede, D.M. Examination of abiotic cofactor assembly in photosynthetic biomimetics: site-specific stereoselectivity in the conjugation of a ruthenium(II) tris(bipyridine) photosensitizer to a multi-heme protein. *Photosynth Res* **2020**, *143*, 99-113, doi:10.1007/s11120-019-00697-8.
